# Collaborative block design task for assessing pair performance in virtual reality and reality

**DOI:** 10.1016/j.heliyon.2020.e04823

**Published:** 2020-09-14

**Authors:** Valtteri Wikström, Silja Martikainen, Mari Falcon, Juha Ruistola, Katri Saarikivi

**Affiliations:** a Cognitive Brain Research Unit, University of Helsinki, Helsinki, Finland; b Glue Collaboration, Fake Production Ltd, Helsinki, Finland

**Keywords:** Psychology, Block design, Collective intelligence, Pair performance, Virtual reality, Social Computing, Computer-supported cooperative work

## Abstract

Collaborative problem solving is more important than ever as the problems we try to solve become increasingly complex. Meanwhile, personal and professional communication has moved from face-to-face to computer-mediated environments, but there is little understanding on how the characteristics of these environments affect the quality of interaction and joint problem solving. To develop this understanding, methods are needed for measuring success of collaboration. For this purpose, we created a collaborative block design task intended to evaluate and quantify pair performance. In this task, participants need to share information to complete visuospatial puzzles. Two versions of the task are described: a physical version and one that can be completed in virtual reality. A preliminary study was conducted with the physical version (N = 18 pairs) and the results were used to develop the task for a second study in virtual reality (N = 31 pairs). Performance measures were developed for the task, and we found that pair performance was normally distributed and positively associated with visuospatial skills, but not with other participant-specific background factors. The task specifications are released for the research community to apply and adapt in the study of computer-mediated social interaction.

## 1 Introduction

Collaborative problem solving is a vital future skill. For instance, in professional contexts, the problems that people focus on are growing in complexity, and finding optimal solutions requires input from multiple people. Because of this, it has been recognized that there is a pressing need for methodologies for measuring and supporting the development of collaborative skills in various environments. (Fiore et al., 2018)

At the same time as the need for collaborative problem solving grows, it increasingly takes place online. Investigating the effects of online environments on collaborative problem solving is an important field of inquiry, as the tools used for collaboration can have profound effects on the individual and shared cognitive processes that determine the success of joint problem solving. This research requires valid methods that work in various problem solving contexts, are comparable, and enable quantifying the success of collaboration.

While trying to find collaboration tasks to use in our own research, we identified a need for general, well-documented tests for measuring cooperational success, so that technological augmentations and other methods for improving social interaction in both virtual and physical environments could be evaluated quantitatively. In this paper, we present a block design pair problem solving task that can be used to evaluate the performance of pairs in different controlled situations. The task and all necessary materials are released as open source^1^ for the community to use and are designed to work in both physical reality and virtual reality (VR).

The aims of this study were (1) to create a collaborative task, (2) to perform a preliminary study of the task with a physical version, (3) to create a VR version of the task based on the outcomes of the preliminary study, (4) to study whether task difficulty is adequate for investigating differences in joint problem-solving performance between pairs, and (5) to study to what extent participants' individual background factors and visuospatial abilities explain performance in the task. We found that the task we created was engaging for participants, collaborative, and functional both in VR and reality. Additionally, the puzzle difficulty produced adequate variance for studying performance, and pair performance was associated with visuospatial skills of the individuals, but not with other background factors.

## 2 Background

### 2.1 Testing cooperation

Collaborative problem-solving in online environments has previously been investigated using a very wide variety of tasks. These include traditional cognitive tests such as matrix reasoning and memory tasks (Chikersal et al., 2017; Engel et al., 2014), creative discussion and production (Siddiq and Scherer, 2017), business cases (González et al., 2003), an electronics problem (Andrews-Todd and Forsyth, 2020), and visuo-spatial tasks (Shen et al., 2018; Heldal et al., 2005). The tasks used differ in many respects, including the extent of actual collaboration required to solve the problem, the measurability of success, the method for determining success in the task, and the specific types of skills and knowledge required.

Especially the tasks used in studies by Chikersal et al. (2017); Engel et al. (2014); Siddiq and Scherer (2017); Buisine et al. (2012); González et al. (2003); Heldal et al. (2005), while used to study cooperation, do not necessarily demand interaction and joint problem solving. This means that a single highly skilled individual could potentially complete the task alone and perform well without input from the other team members. Hence, success in these types of tasks might not always represent factors related to the quality of cooperation. The need for cooperation can be increased by manipulating what information is presented. For instance, in simpler collaborative online tasks, such as the deduction tasks in Care et al. (2015), the amount of information each participant sees is manipulated to increase communication during the task. We will refer to tasks where both participants have the same amount of information as symmetrical, and tasks where the amount or quality of information is different as asymmetrical.

**Figure 1:**
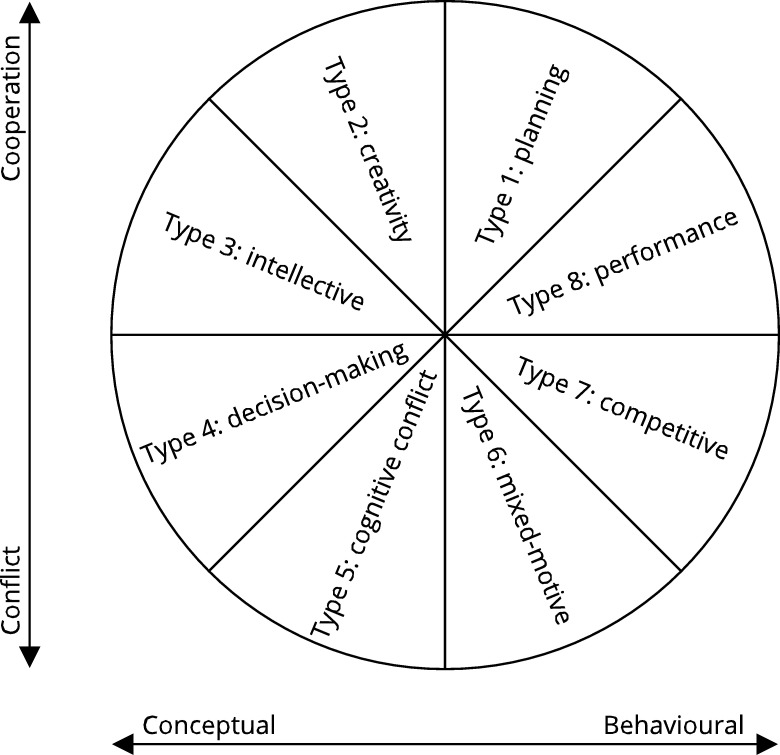
The group task circumplex model developed by McGrath (1984) classifies group tasks into 8 types on a two-dimensional scale.

The McGrath (1984) circumplex model is a tool for classifying group tasks (Fig. 1). This model organizes tasks on a two-dimensional scale, one axis being conflict–cooperation and the other axis conceptual–behavioral. Tasks are classified into 8 distinct types based on these axes. Type 1: planning tasks (high cooperation, moderately behavioral) are about generating plans. Type 2: creativity tasks (high cooperation, moderately conceptual) have idea generation as the goal. Type 3: intellective tasks (moderate cooperation, highly conceptual) have a correct answer. Type 4: decision-making tasks (moderate conflict, highly conceptual) do not have a single right answer. Type 5: cognitive conflict tasks (high conflict, moderately conceptual) are about resolving conflicts of viewpoint. Type 6: mixed-motive tasks (high conflict, moderately behavioral) are about resolving conflicts of interest. Type 7: competitive tasks (moderate conflict, highly behavioral) consider tasks in which participants compete to win. Type 8: performance tasks (moderate cooperation, highly behavioral) are scored tasks which are performed against a standard of excellence.

Hidden profile tasks are tasks in which participants are given different background information, and the best solution can only be reached if the participants successfully combine their complementary information (Stasser and Titus, 2003). This type of task was introduced by Stasser and Titus (1985) in a paradigm where participants were given information about political candidates, so that the information given to each participant would favor a different candidate than the shared information between all participants, and the combined information of all participants would be needed to reach an optimal solution. Participants were asked to discuss the candidates and choose the most suitable one. It turned out that participants typically failed to choose the most suitable candidate based on the combined information, and instead ended up with the candidate that was favored by the majority of participants' individual information or by the common information given to all participants (Stasser and Titus, 1985).

Similar tasks have been used to study success in computer-mediated communication, such as the murder mystery task used by Balakrishnan et al. (2008) to study the effect of information visualizations, the personnel selection task used by Brich et al. (2019) to study working memory support functionalities on a multi-touch table, and the student selection task used by Hassell and Cotton (2017) to study the effect of viewing your own video in video-mediated communication. This type of hidden profile task relies on large amounts of verbal or textual material, and it was originally developed to study bias in group decision making, not group performance across conditions. Because of the complex nature of information, these tasks are usually not symmetrical, in the sense that the information given to each participant differs qualitatively. Reflecting on the McGrath (1984) group task circumplex, these tasks are best represented by Type 4 (decision making), containing elements of Type 5 (resolving conflicts of viewpoint). Due to the distribution of relevant information, hidden profiles have a clear requirement for cooperation, but for a performance measuring task, we are especially interested in Type 3 (intellective) and Type 8 (performance) tasks: cooperative tasks with a clear solution or performance measure.

A task created by Henning et al. (2001) requires two participants to simultaneously coordinate an object through a maze with separate joysticks in a situation resembling a video game. In this symmetrical Type 8 (performance) task, collaboration is required for pacing the object's speed and movements, but the collaboration is dependent on visually observing the movements of the object and no additional information sharing is required. In a two-person puzzle task presented by Kraut et al. (2002) a pair is formed with “helper” and “worker” roles. The helper of the pair gives the worker instructions on how to build a simple puzzle based on the model that the helper, but not the worker, can see, while both team members are viewing separate computer screens. This asymmetrical Type 3 (intellective) task does not require two-way information sharing, as only one member of the pair knows how the completed puzzle should look like.

Traditional intelligence test batteries for assessing individual performance, such as the Wechsler Adult Intelligence Scale (WAIS) (Wechsler, 2005, Wechsler, 2014), typically contain a block design test, which is usually a variation of the Kohs (1920) test. In these tests, the participant is given a task to arrange colored blocks to match a target image and the performance is scored based on correct assembly and speed. Within the WAIS test battery, the block design subtest is shown to have a high correlation with the overall performance on the whole scale (Wechsler, 2005) and can therefore be considered a valid measure of cognitive ability in and of itself. It is still not clear how well visuospatial group problem solving tasks would predict groups' overall collective intelligence, and it is likely that different types of tasks are needed for assessing different aspects of group problem solving. However the use of group tasks that are comparable to validated individual problem solving tasks allows for controlling the effect of individual skills when measuring group problem solving ability, and thus makes it possible to study other environmental and individual factors that potentially play a role in the problem solving situations.

### 2.2 Technology for improving collaboration

One of the big promises of VR technology is known as the *metaverse*, a virtual social world where people can interact with each other in an environment where virtually everything is possible (Bailenson, 2018). The promise of social VR is evidenced by the success of VRChat, a platform for creating social virtual environments, which at an early access stage has become one of the most popular VR applications on the Steam store (Valve Corporation, 2019). An advanced “consensual hallucination” was imagined already by Gibson (1984) in his acclaimed novel *Neuromancer*, at a time when head-mounted displays and early immersive video applications were first being developed at research laboratories (Sutherland, 1968; Lippman, 1980; Chung et al., 1989). In Gibson's “cyberspace” users are connected to the networked computer system directly through a neural interface, capable of stimulating all senses.

Even though the last decade has seen a rapid development of VR technology, we are still far from Gibson's neural interface now, 35 years later. Contemporary commercial VR has apparent expressive limitations, such as the considerably hefty headset covering the users eyes, making facial expression detection difficult (Olszewski et al., 2016), limited haptic feedback to simulate the sense of touch and the detection of the user's body limited to the head and hands (Anthes et al., 2016). These may be just temporary problems, as the technology is rapidly evolving and getting adopted by users. On the other hand, VR is especially intriguing from a computer-mediated communication perspective because it is not limited to trying to mimic reality and face-to-face communication, instead offering the possibility for an *enhanced* environment where any aspect can be tweaked and augmentations to optimize communication can be made with relative ease.

A lot of such augmentations for improving both face-to-face and computer-mediated communication have been proposed over the years, for example hearing (Janssen et al., 2010), feeling (Lotan and Croft, 2007) and seeing (Dey et al., 2017) another person's heartbeats, and enhancing the smiles of facial expression detecting avatars (Oh et al., 2016). The resulting improvements have been investigated with questionnaires and through behavioral aspects, such as inter-personal distance or positivity of language used. In studies of face-to-face collaboration between adults, as well as children, interventions for improving collaboration have been investigated. Preceding interaction with joint rhythmic activities such as rocking in rocking chairs in the same rhythm (Valdesolo et al., 2010), clapping in synchrony (Hove and Risen, 2009), bouncing in synchrony (Cirelli et al., 2014) and playing a musical game together (Kirschner and Tomasello, 2010) have resulted in improved joint action, increased affiliation, altruism, and spontaneous helping behavior. In most of these cases the improvements have been evaluated qualitatively or through a tangential behavioral measure, but reports of quantitative improvements in collaboration are limited. Our aim in this study is to develop a task for specifically this need, a controlled task with a performance measure and scoring system.

## 3 Requirements for a collaborative task

To create a widely useful, general and truly collaborative task, we started off by defining a set of requirements. These requirements were motivated by the aim of generating a task with no clear predefined leader-follower roles, instead giving the participants the option of taking on such roles based on natural social interaction. Since the purpose was to create a useful and comparable task for a variety of situations, we wanted to limit the task's dependence on written information, making it more comparable across different languages and easier to translate. Finally, we wanted to avoid a situation where the task could be completed by a single participant without collaborating with others.

Based on these foundations, we defined three principal requirements:

1. The task should be symmetrical, so that all participants are essentially completing the same task and do not have distinct roles or different subtasks. This requirement ensures that each participant has an equal opportunity to contribute to the end result and that natural interaction can determine the kind of behavior and roles that each participant adopts.
2. To be useful and comparable across studies, the task should be based on simple rules that can be easily adapted to different languages and that are understandable to participants from various backgrounds. This requirement ensures that studies can be performed and compared in a wide variety of situations and across cultures.
3. All team members' contributions must be necessary for completing the task. Each team member should possess some information or skill that is needed and has to be shared with other members for success. This is important for the task to be truly cooperative, because a task that can be completed alone does not necessarily require any type of collaboration during completion.

In addition to these general requirements, we wanted to create an expandable task that cannot be learned from one attempt and that has measurable outcomes to allow for quantifying collaborative success. Furthermore, we wanted to create a task that could be implemented both face-to-face and in VR. The networked VR environment, which we are using for this task, allows for a group of people to communicate through speech and exists as avatars in a shared space, and to manipulate objects in that space. For this reason, a task which involves three-dimensional manipulation of objects became the most apparent direction. Finally, we decided to focus on a pair problem solving task, which could later be expanded for larger teams if necessary.

## 4 The task

To fulfill the requirements listed in the previous section, we designed and developed a novel collaborative block design task. The task consists of spatial configuration puzzles built with a set of three-dimensional blocks. The set consists of seven different shapes and two different colors, for a total of 14 blocks. No duplicate blocks of the same shape and color are used. The shapes included are cube, cylinder, sphere, pyramid, cone, long cuboid and long cylinder. These shapes are shown in red color in Table 1. The most important feature of the shapes is that each of them resembles at least one other shape from each flat two-dimensional side projection. In other words, the shapes are not unambiguously identifiable from only one flat image. This design choice allows for the creation of puzzles that require two different viewpoints to be solved.

**Table 1:** Each shape included in the task. In the table, the three-dimensional shapes are depicted in the left column and flat two-dimensional projections of the shapes from two different views in the middle and right columns.

	3D render	View 1	View 2
Cube			

Cylinder			

Sphere			

Pyramid			

Cone			

Long cuboid			

Long cylinder			

**Figure 2:**
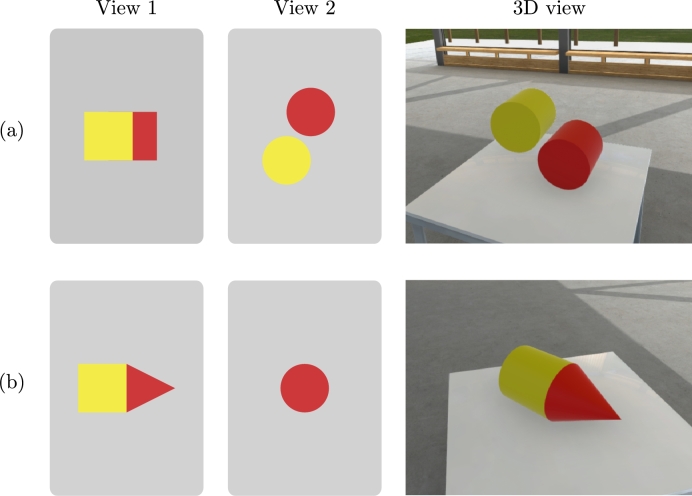
Example puzzles featuring partial (a) and complete (b) occlusion of blocks, as well as flat projections of three-dimensional shapes.

Each puzzle consists of two cards with flat two-dimensional images and a target three-dimensional configuration. The configuration is such that each of the blocks is at a right, orthogonal angle with every other block, meaning that they can only be rotated 90 degrees at a time. The direction of the cards (which side is up), or the side of the projection in the cards (from which angle the card is depicting the target configuration) is not specifically determined and the target configuration can be reached in any rotation. In other words, the participants can freely choose to build the configuration sideways or upside down compared to how the puzzle designer planned it. Blocks can be partially or completely occluded by other blocks in the cards (as in Fig. 2), but no blocks that are completely occluded in both cards are part of the target configuration.

During a puzzle, each participant receives one card depicting the same target configuration, but from different angles. Participants are not allowed to show their cards to each other. To solve the task, they have to communicate the contents of their cards to each other either verbally, non-verbally or using the blocks and reach a mutual conclusion regarding the target configuration. Other aids, such as pen and paper, are not allowed. Once the participants are satisfied with their solution, the puzzle ends and the correctness of the solution can be determined.

The standard version of the task does not limit the amount of time that the participants can use for solving each puzzle. The task is designed to also work with puzzle-specific time limits dependent of the desired failure rate. This version requires puzzles with validated mean completion times.

Most puzzles are specifically designed to be unambiguous, so that there is only one possible target configuration for a set of two flat images. In other words, each set of two flat images should only result in one specific three-dimensional configuration, even though many configurations allow the participants to freely determine whether the cards are depicting the configuration from the sides, the top, or even the bottom. The motivation for this is that difficulty of puzzles that have multiple solutions can be problematic to determine: reaching one solution might be easier than another. During the development of the task, we noticed that it can be quite difficult to determine, without user testing, whether a puzzle has multiple solutions. As a result, some tasks included in the study turned out to have a second solution that had not been apparent to the puzzle designer (see Supplementary Appendix A).

### 4.1 Administering the test

In the beginning of the task, the participants are familiarized with the blocks with the help of the pictures depicted in Table 1. The administrator explains that seven different shapes exist in two different colors, for a total of 14 blocks. The participants are briefed on the concept of flat two-dimensional projection, explaining that the flat projection does not show details or shadows, such as the tip of the pyramid shape, but rather the outline of the shape from a certain orthogonal angle. The administrator confirms that the participants understand the concept of flat two-dimensional projection, and if not, the explanation is repeated until the participants confirm that they understand it.

Next, the administrator explains that each puzzle consists of two or more blocks and that the target configuration is represented with the flat two-dimensional projection from two different angles in two cards. The participants are told that the angles do not matter, that the card can be rotated any way they like, and that they can decide from which sides they are seeing the cards. In the case of the physical version of the task, the administrator tells the participants that during the task, they are not allowed to show the cards to each other. In the VR version, the cards are represented as wall posters, and the participants are told that each card is only visible to one participant. The participants are told that they are free to discuss the contents of the cards and to use the blocks as communication aids.

At this point, an example puzzle is presented by showing two cards so that they can be seen by both participants. The example puzzle is chosen so that it features partial occlusion of one of the blocks, as well as flatness of a three-dimensional shape, such as in Fig. 2 (a), with the cylinder's side represented as a flat rectangle and one of the cylinders occluding the other. The administrator solves the task while explaining the motivation for placing the blocks in the target configuration. The administrator explains which angles are represented in each card and how the occlusion is depicted. The participants are asked whether they understand the task structure and the concepts of occlusion and flat projection and the explanations are repeated until they confirm that they do.

Next, a second example puzzle is shown, once again so that both cards can be seen by both participants. The second example should include complete occlusion of one of the shapes, such as in Fig. 2 (b), with the cylinder completely occluded by the cone from one angle. This time the participants are asked to solve the puzzle by themselves and told that they can freely ask clarifying questions from the administrator during this example task. Once participants are finished, the puzzle and complete occlusion of the cylinder is explained and a possible incorrect solution is corrected.

The participants are instructed that their goal during the task is to solve puzzles as accurately and as quickly as possible and that they need to determine when they are finished with a task and to communicate this to the administrator. At this point the administrator asks the participants if they understand the rules of the task and if they have any questions before starting the task, during which the administrator can not answer any questions about the puzzles. If the participants have no further questions, the task begins.

The task consists of consecutive puzzles. Each puzzle begins with one card being dealt to each participant in the physical version or one wall poster shown to each participant in the VR version. Time measurement begins when the puzzle-specific pictures are shown to the participants. Participants solve the puzzle without distraction or additional assistance from the administrator and once they are finished, the completion time is recorded. Finally, the administrator inspects the configuration reached by the participants and records whether it is correct or not. Participants are not given feedback on the correctness of their solutions during the task, but feedback may be freely given after all the puzzles and any additional tests and questionnaires have been completed.

### 4.2 Physical version

A physical set of blocks was manufactured for a user study to assess completion times and subjective experience of users (see Section 5). The blocks depicted in Table 1 were 3D printed, in sizes of 2 x 2 x 4.4 cm and 2 x 2 x 2 cm, for the elongated shapes and all the other shapes respectively. Two sets of seven blocks were 3D printed in PLA plastic. Cards were printed in color on cardboard, including shape cards represented in Table 1, example task cards in Fig. 2 and task puzzle cards in Supplementary Appendix A. The backs of the cards were labeled by hand to make administration of the task easier.

**Figure 3:**
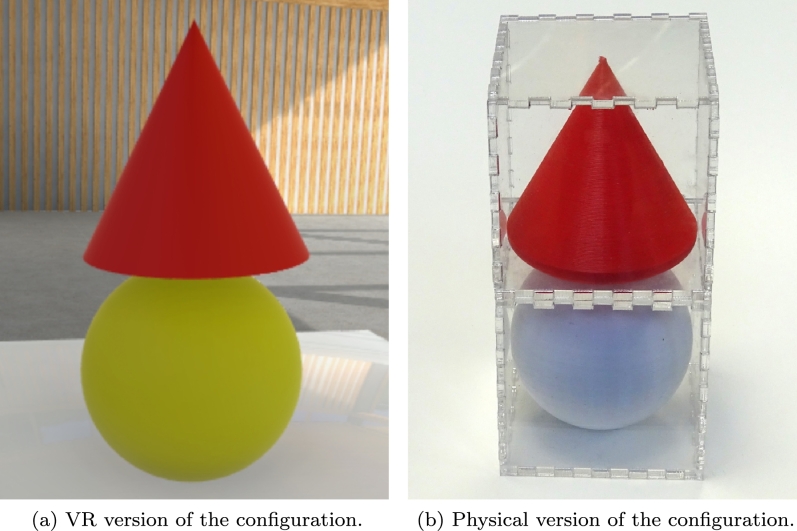
An example of a configuration of blocks which would not be possible to create in physical reality without the support structure provided by the acrylic enclosures.

Limitations of the physical reality do not allow for certain configurations of three-dimensional shapes to be stacked on top of each other, in comparison to VR where blocks can be left suspended in air and without a stable base. This limitation was alleviated by enclosing the blocks in regular sized cuboid-shaped transparent acrylic containers, so that all blocks could be placed on top of each other (Fig. 3). The acrylic containers were manufactured with an Epilog Legend 36EXT laser cutter. The acrylic material was 2 mm thick and the inner dimensions of the containers were matched with the block sizes, leading the outer dimensions to be 2.4 x 2.4 x 4.8 cm for the long cuboid and cylinder blocks and 2.4 x 2.4 x 2.4 cm for all the other blocks.

### 4.3 Virtual reality environment

The VR experiment was performed in a commercial software system specifically designed for collaboration in virtual environments. The system consists of a client software application and a server software application. There is one running server application instance maintaining the shared simulated state of each virtual environment, and one running client application instance for each user. The client collects inputs from the user and forwards them as network messages to the remote server over a local area network connection or over the public Internet. As messages containing user inputs arrive from each client, the server validates the inputs according to the rules of the simulated environment, updates the simulation state, and sends a response to all clients. The clients then process the state changes described within the response and update their local representation of the shared simulation accordingly. In cases of conflicting representations, the server version is considered correct.

Each user wears a VR headset and a controller in each hand. The position and orientation of both the headset as well as the controllers are tracked by the VR hardware and software systems, in this case consisting of HTC Vive and SteamVR. The tracked data is sent to the server which then updates the position and orientation of the virtual representations of each user's head and hands. These representations or avatars are considered part of the shared simulation and each client application therefore has a visual representation of all other participants within the virtual environment.

User voice is also captured using a microphone and shared across the network. Each user wears headphones and the client application simulates spatial sound point sources, therefore simulating the voices of remote users originating from the positions of their corresponding virtual representations.

#### 4.3.1 The task in VR

**Figure 4:**
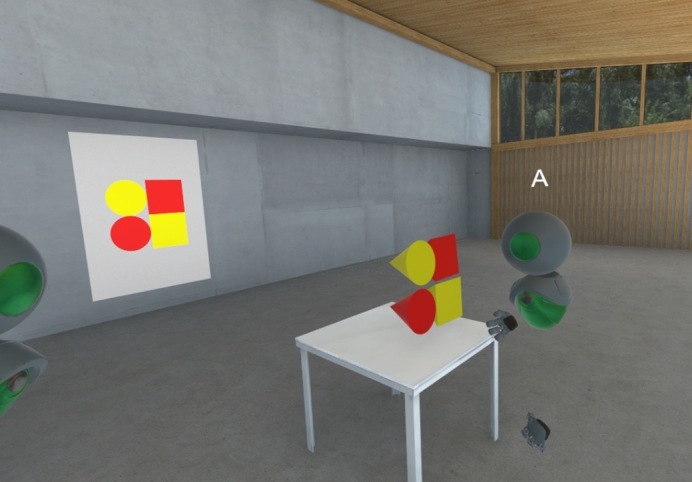
Screen capture of the virtual environment used in the second study.

For the experiment, a private wired local area network was configured to ensure predictable networking conditions. A virtual scene was configured in the VR environment, consisting of a sufficiently large room to contain all the required 3D objects for the task, two sets of 3D objects representing the blocks, and a set of 3D objects representing the shape cards (Fig. 4).

The block sets consisted of one yellow set and one red set of the same seven shapes used in the physical version of the task. After preliminary testing showed that white colored objects were sometimes difficult to perceive in the 3D environment, the color of the white blocks was changed to yellow compared to the physical version. The users were able to grab the blocks using the handheld controllers and freely move and rotate them within the virtual environment. No gravity or collisions were simulated, enabling a free form configuration of the shapes, apparently floating in free space. A virtual table was placed in the environment as a purely visual cue for the users to place their solution for the block configuration requested in the task.

The shape cards were all hidden except for one pair at a time. Also, a modification was implemented to the network synchronization algorithm within the collaboration software system. This modification ensured that out of each shape card pair, each participant saw exactly the card which was hidden from the other participant. The local representations on each client were therefore purposefully out of sync for the purpose of the experiment. After the completion of each puzzle, a feature of the collaboration software system was used by the administrator to reset the environment, which automatically returned the blocks back to their initial positions in the environment. The administrator then selected a new pair of visible shape cards for the next puzzle attempt. The ordering of the puzzles was consistent for all participants.

## 5 Preliminary study

A series of two studies were performed. The aim of the preliminary study, performed with the physical version of the task, was to answer the following research questions to inform the design of the second VR-experiment:

• RQ1: Is the task engaging and does it produce collaboration?
• RQ2: What is the difficulty level of the individual puzzles?

Sets of five puzzles were designed for the preliminary study, and each pair of participants performed either set 1 (10 pairs) or set 2 (8 pairs). Set 1 consisted of Puzzles 1-5 and set 2 consisted of Puzzles 6-11. One item of set 2 (Puzzle 9) was excluded for being too difficult, with only one of the first four pairs being able to solve it, and it was replaced with Puzzle 11 for the consecutive four pairs completing set 2 (the fourth pair completed both Puzzles 9 and 11).

We hypothesized that the amount of pieces needed for completing a puzzle would have a positive correlation on task difficulty, and because of this we aimed to balance the puzzles so that the puzzles in each set would have a variable amount of pieces. To gather comparable difficulty data between the puzzles, the order of the puzzles within each set was randomized for every pair completing the study, as well as the order in which the participants were given the two different cards for each puzzle.

### 5.1 Methods

#### 5.1.1 Participants

The participants for the preliminary study were recruited at public places around local university campuses. 36 participants (15 women, mean age = 28.8 years, standard deviation (SD) = 4.2 years) participated in the preliminary study as 18 dyads (16 same gender pairs and 2 mixed gender pairs).

#### 5.1.2 Procedure

Before the task, participants were asked to fill out a standard demographic background questionnaire on paper, which included questions on age, socioeconomic status, and gender. After this, the task administrator followed the protocol outlined in Section 4.1 using the physical version of the task components described in Section 4.2.

After the participants announced that they had finished a puzzle, the completion time and correctness of solution were evaluated. To limit the maximum total time required to finish the whole task to 45 minutes, if participants had not reached a conclusion on a puzzle within 540 seconds, the puzzle was concluded by the administrator and recorded as incorrect. After each puzzle, the participants were asked to answer individually, without consulting each other, the Single Ease Question (SEQ), which is a subjective easiness rating ranging from 1 (very difficult) to 7 (very easy), that has been shown to perform equally well or better than more complicated metrics (Sauro and Dumas, 2009). After the set of five puzzles had been completed, the participants were asked to individually complete the NASA Task Load Index (NASA-TLX) set of six questions (Hart and Staveland, 1988) to assess perceived mental and physical load, perceived time demands, performance, effort and frustration. Finally, a short open-ended interview was performed for 9 pairs (which were all same-gender pairs) to gather subjective ethnographic data of the experience.

## 6 Results of the preliminary study

To assess the difficulty of the task, completion times, amount of correct answers, and the SEQ were analyzed. Additionally, participant responses to short interviews conducted after the task were examined with a focus on experiences related to engagement and collaboration.

### 6.1 Completion times and perceived difficulty

**Figure 5:**
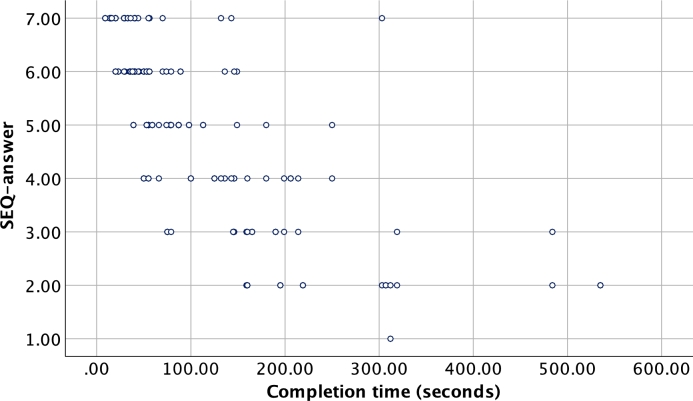
Association between completion times and SEQ answers (1 - very difficult to 7 - very easy) for the puzzles in the physical version.

The mean completion times obtained in the preliminary study are shown in Table 2. Based on the obtained completion times and SEQ answers, the puzzles were balanced into two similar difficulty sets for the VR experiment, taking into account also the amount of blocks needed to complete each puzzle. Fig. 5 shows the association between completion times and SEQ answers in the preliminary study.

**Table 2:** Mean completion times and SEQ values for puzzles in the physical version. Only correctly completed puzzles were used in calculating the finishing times.

VR puzzle	Physical puzzle	Blocks needed	Pairs	% correct answers*	Mean finish time (sd)	Mean SEQ (sd)
1.1	Puzzle 7	2	8	100	34.4 (17.9)	5.9 (1.0)
1.2	Puzzle 2	2	10	100	70.4 (46.8)	5.8 (1.4)
1.3	Puzzle 8	3	8	62.5	125.0 (112.3)	3.3 (1.9)
1.4	Puzzle 4	3	10	70	168.3 (98.3)	3.9 (1.2)
1.5	Puzzle 10	4	8	87.5	165.3 (26.3)	3.6 (1.3)
2.1	Puzzle 6	2	8	100	40.5 (29.3)	6.2 (1.1)
2.2	Puzzle 1	2	10	100	44.4 (23.8)	6.3 (0.8)
2.3	Puzzle 11	3	5	100	77.4 (11.5)	4.5 (1.1)
2.4	Puzzle 3	4	10	100	138.0 (64.0)	4.6 (1.7)
2.5	Puzzle 5	4	10	70	260.1 (151.8)	3.2 (1.7)
–	Puzzle 9	4	4	25	484.0 (−)	1.4 (0.7)

* The puzzle was concluded and marked as incorrect if completion time was 540 seconds or longer (for 2 pairs with Puzzle 5, 2 pairs with Puzzle 8, and 2 pairs with Puzzle 9).

### 6.2 Subjective experiences

The short interviews that were conducted in the preliminary study, aimed at obtaining open feedback from participants, revealed that all interviewed pairs felt that they were collaborating during the task. Three pairs spontaneously remarked that they learned to communicate better during the task, either by adopting a new strategy, or realizing something about the other person's way of thinking, which helped in solving the puzzles. At least one person from each pair spontaneously remarked that the task was fun, which was also reflected in that all participants seemed engaged during the task, even the ones that were initially negative, or hesitant to participate, as evaluated by the researchers conducting the task.

### 6.3 Task development

The 10 puzzles from the preliminary study were used to form two sets of puzzles with equal difficulty for the VR version of the task. For this version, in both sets the individual puzzles were ordered by their difficulty and number of blocks needed from the easiest to the hardest and named SET 1: puzzles 1.1 to 1.5 and SET 2: puzzles 2.1. to 2.5 (see Table 2).

## 7 Second study

The aim of the second study, performed with the VR version of the task, was to answer the following research questions:

• RQ3: Is the task adequately difficult to produce variance in pair performance as measured by completion times?
• RQ4: Does the perceived task load (as measured by the NASA task load index) differ from the physical version?
• RQ5: Is pair performance predicted by individual background factors such as age and gender, and task-related factors such as the order in which the sets are presented?
• RQ6: Is pair performance predicted by individual visuospatial abilities?

### 7.1 Methods

#### 7.1.1 Task order and difficulty

In the second study the puzzles within each set were always completed in the same order, and the division of pair cards were kept the same within a pair. Only the order of the two sets were randomized between pairs. The motivation behind this study design, and behind creating two sets with similar difficulty, was to allow for controlled trials where the two sets can be used in testing different conditions. All pairs completed both sets of puzzles. Half of the pairs (N = 16, 51.6%) were randomly selected to complete SET 1 first, and the order was reversed for the rest. An individual puzzle was concluded if pairs exceeded a 900 second time limit in trying to solve it. We chose to have a longer time limit in the second study to avoid a potential floor effect as the VR environment was unfamiliar for the participants and thus might slow down the problem solving.

#### 7.1.2 Participants

The participants were recruited via social media and the university's student mailing lists. Participants were right-handed, Finnish-speaking adults, and had normal or corrected-to-normal vision. Right-handed participants were chosen due to the requirements of a psychophysiological experiment included in the session, which will be reported elsewhere. The participant pairs did not know each other before the experiment and were only allowed to meet face-to-face after the experiment. Altogether 66 adults participated in the experiment (33 pairs) of which one pair was excluded for knowing each other and one pair was excluded from further analysis due to equipment failure, leading to 62 included participants (31 pairs). The University of Helsinki Ethical Review Board in the Humanities and Social and Behavioural Sciences approved the study protocol. All participants provided written informed consent.

#### 7.1.3 Measures

An online background questionnaire was used to gather data on variables that could influence performance, including age, socioeconomic status, and gender, as well as prior experience with VR. The block design subtest of the WAIS-III test battery was used, to measure visuospatial reasoning skills in order to control for their effect in joint problem-solving success. The subtest was administered following the test guidelines (Wechsler, 2005) by a psychologist or an experienced research assistant supervised by a psychologist and it was completed in a quiet room where only the experimenter and the participant were present prior to the VR experiment.

Completion times in the task were used as a measure of joint problem-solving performance (time from the pair being presented with a new puzzle to the pair stating that the puzzle was completed). Based on the completion times for each puzzle, a scoring system was also created, where pairs were divided into tertiles by the presenting order of the set (SET 1 first or SET 2 first). The fastest tertile in each specific puzzle was appointed 3 points per puzzle, the second tertile 2 points per puzzle, and the slowest tertile 1 point per puzzle. Pairs with incorrect or too slow (> 900 seconds) solutions were assigned 0 points per puzzle. The points were added to indicate the sum scores for all puzzles for each set and pair.

The SEQ was used to assess the difficulty of each puzzle on a scale of 1-7 (1=very difficult, 7=very easy). The ratings were obtained to be able to create sets of tasks that were balanced in their difficulty.

The NASA-TLX was used to assess perceived mental and physical load, perceived time demands, performance, effort and frustration after each set of five puzzles. The NASA-TLX results were used to compare the demands of the physical and the VR version of the task. We used a scale from 1 to 10 instead of the original scale from 1 to 21, with higher ratings indicating higher task load in the specific domain. We calculated a grand mean from one participant's answers after both sets to indicate overall task load.

#### 7.1.4 Procedure

Prior to arrival, participants answered the online background questionnaire. Upon arrival, the participants completed the block design subtest of the WAIS-III test battery (Wechsler, 2005) in separate rooms. After the test, participants were escorted to their respective VR rooms where they were initially familiarized with the VR equipment. This included moving around and manipulating the objects, as well as teleportation and the chaperone grid representing the real physical walls in the VR space.

During the task, completion times were scored by two observers separately, who were assisting the two participants with their VR equipment. Means of the two observers' scores were used to represent the completion times. If the measurement was missing (or marked as ambiguous) from either of the observers, the other observer's individual scoring was used (in 36 out of 310 cases), the mean difference between the ratings was 4.7 seconds (SD = 7.3 s, min = 0 s, max = 64 s). The intraclass correlation between ratings was higher than 0.99 indicating excellent inter-rater reliability (p < 0.001). The mean completion times for each puzzle in both sets were calculated separately based on the set's presenting order (first or second).

In between puzzles, participants completed the SEQ. To be able to report this answer while wearing the VR headset and to make sure that they would not reveal their rating to their pair through the microphone, the participants were asked to show their answers to the assistant using their fingers, who wrote the response down. The NASA-TLX questionnaire was completed on paper by the participants individually after each set of five puzzles.

After the task was completed, the participants were assisted out of the VR gear and got to meet each other face-to-face. Two movie tickets were given as a reward for participation.

#### 7.1.5 Statistical analyses

The significance level was set to two-tailed p < 0.05. To account for inequality of variances and nonnormality of the completion time and task load data, Welsh's T-tests with bootstrap estimated p-values were used for comparing means (using 2000 bootstrap samples) (Delacre et al., 2017). Spearman correlations were calculated for assessing associations between SEQ answers and completions times.

We used multiple linear regression analyses to study the effects of the potential covariates (visuospatial skills, age, gender, education, and VR experience) that might affect the pair performance. For education, pairs where both members had a Bachelor's degree or higher (n = 14) were compared to other pairs (n = 17). For VR experience, pairs where both members had at least tried VR 1 to 3 times before (n = 16) were compared to other pairs (n = 15). Three different models (Y = $\beta_{0}$ + $\beta_{1}$ × $X_{1}$ + ... + $\beta_{6}$ × $X_{6}$ + *ϵ*) were tested, where Y = pair's task performance, $X_{1}$ = the pair's average (model 1), lowest (model 2), or highest (model 3) visuospatial reasoning raw score, $X_{2}$ = age of the first member, $X_{3}$ age of the second member, $X_{4}$ pair's gender (1 if same gender, 0 otherwise), $X_{5}$ = education (1 if both have at least Bachelor's degree, 0 otherwise), $X_{6}$ = VR experience (1 if both have tried VR, 0 otherwise).

## 8 Results

### 8.1 Participants

The mean age of the included 62 participants was 30.4 years (SD = 6.4 years). 38 of the participants were women (61%), with 11 female (35%), 4 male (13%), and 16 mixed-gender (52%) pairs. 18 (29%) of the participants had completed high school or vocational education, 22 (35.5%) had a Bachelor's degree, and 22 (35.5%) had a Master's degree or higher. Mean standard score in the WAIS block design subtest was 12.6 (SD = 2.8, min = 5, max = 19) indicating that the participants had better than average visuospatial skills as compared to the Finnish norms (Wechsler, 2005). 29 % of participants were using VR for the first time in the experiment, 53 % had tried VR 1–3 times before, 15% had used VR several times before, and 3% reported that they used VR frequently.

### 8.2 Completion times and sum scores

Table 3 shows the mean completion times for correctly completed puzzles in the VR experiment, based on the presenting order of SET 1 and SET 2, percentages of correct answers, and p-values for the differences between completion times when the same set of puzzles was presented first or second. Pairs managed to solve most of the puzzles without errors. Mean completion times varied from 42 (puzzle 1.1) to 339 (puzzle 2.3) seconds, the standard deviation of completion times was fairly large, and it appears to be dependent on the mean completion time. Completion times were generally longer for the set of puzzles that were presented first, significant differences were found for puzzles 2.3, 2.4, and 2.5 (p-values < 0.044), indicating that the learning effect was more pronounced for puzzles in SET 2 (Table 3).

**Table 3:** Mean completion times of correctly finished puzzles 1.1–1.5 and 2.1–2.5 for when the set is done first or second and p-values for the time difference. Puzzles 1.1–1.5 were completed first by 16 pairs and puzzles 2.1–2.5 were completed first by 15 pairs out of the 31 pairs who completed both sets. Results were removed from one pair in puzzle 1.2 and one pair in puzzle 2.1 due to technical problems during completion of the puzzle.

VR puzzle	Correct answers 1st round*	Mean finish time 1st round (sd)	Correct answers 2nd round*	Mean finish time 2nd round (sd)	Mean difference	p (two-tailed)
1.1	94 %	63.1 (76.4)	100 %	42.3 (14,0)	20.9	0.43
1.2	100 %	62.1 (19.0)	100 %	59.8 (22.2)	2.3	0.76
1.3	69 %	283.6 (234.9)	93 %	202.2 (123.3)	81.5	0.33
1.4	100 %	119.3 (53.1)	100 %	87.1 (29.2)	32.2	0.059
1.5	88 %	376.1 (219.7)	93 %	258.8 (170.7)	117.3	0.12
2.1	100 %	115.6 (73,1)	100 %	84.1 (54.7)	31.5	0.22
2.2	100 %	79.3 (52.4)	100 %	82.6 (151.7)	-3.3	0.94
2.3	80 %	339.0 (215.9)	75 %	170.7 (152.7)	168.3	**0.043**
2.4	100 %	154.0 (52.3)	100 %	105.3 (48.4)	48.8	**0.019**
2.5	93 %	252.1 (141.8)	94 %	144.5 (66.9)	107.6	**0.041**

* The puzzle was concluded and marked as incorrect if completion time was 900 seconds or longer (1 pair for puzzle 1.3, 1 pair for puzzle 1.5, 2 pairs for puzzle 2.3).

Based on the scoring system described in the Section 7.1.3, the sum scores of all puzzles per pair were normally distributed (indicated by the Shapiro-Wilk test, p = 0.161) with a mean of 18.8 points (SD = 4.8, min = 8, max = 27 points, from an overall range between 0 to 30 points).

### 8.3 Perceived difficulty and task load

The puzzle specific mean answers to the SEQ varied from 3.2 (puzzle 2.3) to 6.7 (puzzle 1.1) in the VR experiment and from 3.2 (puzzle 2.5) to 6.3 (puzzle 2.2) in the physical experiment (excluding Puzzle 9 which was replaced with Puzzle 11 during the preliminary study). The mean for all answers for all VR puzzles to the SEQ was 5.0 (SD 4= 1.7) and mean of the correctly solved completion times was 148 s (SD = 146 s), and for the physical puzzles the SEQ mean was 4.6 (SD = 1.9) and the completion time mean was 113 s (SD = 103 s). Spearman correlation between SEQ answers and the correctly solved completion times was −0.79 (p < 0.001, N = 571) for the VR version and −0.79 (p < 0.001, N = 148) for the physical version (Fig. 5).

When comparing the NASA-TLX answers between the physical experiment and the VR experiment (Table 4), significant differences were found for physical demand with a score +1.6 for VR compared to physical (p < 0.001), indicating higher physical demand for the VR version, and for performance with a score +2.7 for VR compared to physical (p < 0.001), indicating a worse performance rating in the VR version.

**Table 4:** NASA-TLX scores for the physical experiment and VR experiment with a scale from 1 to 10.

	VR mean score (sd)	Physical mean score (sd)	Mean diff	p (two-tailed)
Mental demand	4.7 (2.0)	5.5 (2.4)	-0.8	0.099
Physical demand	2.5 (1.5)	0.9 (0.6)	1.6	< **0.001**
Temporal demand	3.4 (1.6)	4.0 (2.0)	-0.6	0.11
Effort	6.1 (1.7)	5.9 (2.3)	0.2	0.65
Performance	5.0 (2.3)	3.3 (1.9)	1.7	< **0.001**
Frustration	3.6 (1.8)	2.9 (2.0)	0.7	0.12

### 8.4 Background factors

Based on the regression analyses described in Section 7.1.5 the pair's WAIS block design mean raw score (unstandardized beta (*B*) = 0.41, 95% confidence interval (CI) = 0.22 - 0.60, p < 0.001), minimum raw score (*B* = 0.31, 95% CI = 0.15 - 0.47, p < 0.001), and maximum raw score (*B* = 0.34, 95% CI = 0.12 - 0.56, p = 0.009) were positively associated with the pair's sum score from all puzzles of the task. Other background variables: gender (p-values > 0.31), education (p-values > 0.07), age (p-values > 0.23) and experience with VR (p-values > 0.16) were not associated with pair performance in any of the three models with either mean, minimum, or maximum WAIS block design raw score as an independent variable.

## 9 Discussion

We created a novel collaborative block design task for pairs, which is best reflected on the McGrath (1984) task circumplex as a Type 3 task, an intellective task with a correct answer, but it also contains aspects of Type 5 (cognitive conflict) and Type 8 (performance) categories. The task's rules are abstract, and the task can be expanded with an arbitrary amount of additional puzzles, and as such it can not be learned during one attempt. The puzzles included in this study were manually created with a vector graphics program; creating a puzzle editor with automatic testing of ambiguous puzzle solutions is an interesting challenge for future work.

We investigated the task's suitability to be used as a collaborative task (RQ1) through a preliminary study completed with a prototype of the physical version of the task and used the task to study the difficulty level of the puzzles (RQ2). The participants of this preliminary study considered the task collaborative, and their behavior reflected positive engagement, as evaluated by the researchers conducting the preliminary study, and as reflected in the open-ended interviews conducted after the task. A similar observation was made by Kohs (1920), the original creator of the one-person block design intelligence test, who remarked that: “It is interesting to watch the response of children and even adults when they are given colored cubes to handle. There is no doubt that an appeal exists which touches the roots of some very fundamental original tendencies.” As such, the block design task is promising, because it seems to be engaging and accessible for most participants. We found that there was considerable variance in the completion times between the different pairs solving the puzzles, suggesting that the task is able to discriminate between different levels of collaborative performance. Both the completion times and subjective experience of difficulty produced similar rankings for the different puzzles, indicating that completion time is a good estimate of subjective difficulty of the task. Based on the preliminary study, we developed two equally difficult sets of five puzzles to test the task in a collaborative VR environment.

In the second study we explored whether the task was adequately difficult to produce variance in pair performance (RQ3) and whether perceived task load differed from the physical version (RQ4). Indeed, based on a scoring system that took into account the presentation order of the puzzles, it seemed that the pair performance was normally distributed and no floor or ceiling effects were observed, indicating a good difficulty level for the task. The task load scores indicated that the task required moderate mental effort and that the participants did not perceive the task especially stressful or frustrating. Physical demand was evaluated as higher, and performance as worse by the participants in the VR experiment than those in the physical experiment. The physical demand score might be explained by the VR experiment requiring standing up and physically moving around, compared to sitting down at a table in the physical experiment. Additionally, wearing a VR headset might be perceived as physically demanding in itself. The lower perceived performance in VR is harder to explain. One reason may be that 82% of participants had a total of three or less VR experiences prior to participating in the study, which could have caused the participants to be insecure about their performance in the whole task.

We also wanted to know whether pair performance was predicted by individual background factors, and task-related factors (RQ5). To investigate possible learning effects, half of the participants completed SET 1 before SET 2, and the order was reversed for the other half. In general, a learning effect could be observed as shorter completion times for the same puzzles when they were presented in the last set. However, when comparing the two sets of five puzzles, we found that the learning effect was somewhat more pronounced in SET 2: 3 out of 5 puzzles were completed significantly faster when they were presented after SET 1, when no significant differences were observed in SET 1. This was also reflected in the performance ratings of the participants. This should be taken into account if the two sets of tasks are used in controlled trials. Background factors of age, gender, education level, and experience in VR were not associated with pair performance.

Finally, we studied whether pair performance was predicted by individual visuospatial abilities (RQ6). In contrast to results by Woolley et al. (2010), who found that group members' individual IQ did not predict group performance in a collaborative test battery, we found that both pair mean, minimum and maximum WAIS block design test scores showed a positive association with pair performance in our task. This effect was largest for the mean scores. This indicates that when controlling for individual visuospatial abilities, the task can be used to study other, interpersonal and environmental, factors of interest that might further predict pair performance. This also means that individuals' visuospatial abilities must be assessed and controlled for when using the task to examine the success of collaboration. However, using a collaborative task in which performance is in part explained by an easily quantifiable and testable individual skill makes the task more useful in assessing factors that influence collaboration than a task that would tap into and depend upon several individual cognitive abilities.

### 9.1 Limitations

As the aim of the preliminary study was to guide the development of the task, rather than to act as a comparison for the VR version, the studies were conducted differently in terms of presentation order of the puzzles and participant selection. Therefore the results of the two studies are not entirely comparable. Especially, the completion times between the two studies cannot be compared to each other, whereas the differences between the two studies are probably less likely to affect the task load answers that regard the test more broadly as a whole. In addition, it can be argued that the difficulty order of the individual tasks can still be evaluated reliably. It should also be noted that the second study only included right handed participants, which might be a source of bias, and that the participants were all relatively highly educated and had good reasoning skills which might compromise the generalizability of the results.

### 9.2 Future research

These results should be validated with a generally larger sample and a more diverse population of participants representing different backgrounds and also including both right and left handed participants. It should also be studied in more detail, how individual skills and experience with similar tasks may affect pair performance. Interesting future research could also focus on how different aspects of the environment affect the performance in the task, and especially how task performance relates to other performance measures, which would add to the predictive validity of the method. The task can also be used in studies assessing different methods and interventions to support collaboration both face-to-face and in VR.

Physical and personality traits, mood, and skills such as empathy might differently influence experience of collaboration in different environments. For instance, similarity in physical appearance has been found to support emotion contagion and empathy in face-to-face situations (De Waal and Preston, 2017). This means that physical dissimilarity may weaken the experience of connection face-to-face, but that in VR, the experience could be different due to avatars appearance being customizable and independent from the physical appearance of the users. In addition to avatar appearance, introducing additional information, from e.g. the users' physiological states has been proposed as a possible way to improve understanding (Janssen et al., 2010; Lotan and Croft, 2007), and quantifiable tasks are necessary to evaluate their efficiency in both virtual and physical reality. When the task is simple, standardized, and the contributions of individuals' skills to collaborative success are known, comparing individual experiences in different environments is more reliable. This also applies to testing aspects of the environment itself, such as how other people are represented in VR as avatars, and how the environment could be better customized (e.g. lighting, design) for different users' needs.

It should be noted that joint attention (individuals actively sharing their focus on the same object and with each other) and active perspective-taking required in this task may by themselves have positive effects on performance. Especially in visual tasks, it has been suggested that actively sharing attention to the same object with another viewer enables perspective sharing that can improve processing of three-dimensional objects (Böckler et al., 2011). There is also evidence that the empathy skills of team members and other empathy-related mechanisms predict the success of collaborative problem solving (Woolley et al., 2010; Chikersal et al., 2017; Engel et al., 2014), and that perspective-taking activity improves trust between collaborators in virtual environments (Erle et al., 2018). Examining the effects of activities that require perspective-taking and joint attention on the success of collaboration is an important future question.

## 10 Conclusion

We have presented a novel joint problem solving task, designed to evaluate pair performance in both virtual reality and reality. Compared to previous tasks used for assessing team interaction, our task is symmetrical, requiring two-way information sharing and collaboration between participants to be completed, is not dependent on the language being used, and it is designed to quantify pair performance. The results of the study are favorable for the intended purpose, including variability in the difficulty of puzzles, similarity of subjective difficulty ratings and completion times, as well as participant engagement.

When controlling for individual visuospatial skills, the task can be used to investigate differences in collaboration between pairs, to find high and low performing pairs, as well as to investigate quantifiable effects of the environment and digital tools on pair performance. We acknowledge that different types of tasks are needed to assess different aspects of pair performance, and the task presented in this paper focuses especially on visuospatial problem solving. Further development of the method should also include investigation of whether performance in the task is connected to success of collaboration in complex real life situations.

The task is released as open source^2^ for the community to use and we encourage others to adapt it to their needs.

## Declarations

### Author contribution statement

V. Wikström, S. Martikainen: Conceived and designed the experiments; Performed the experiments; Analyzed and interpreted the data; Contributed reagents, materials, analysis tools or data; Wrote the paper.

K. Saarikivi, M. Falcon: Conceived and designed the experiments; Performed the experiments; Contributed reagents, materials, analysis tools or data; Wrote the paper.

J. Ruistola: Conceived and designed the experiments; Contributed reagents, materials, analysis tools or data; Wrote the paper.

### Funding statement

This work was supported by Business Finland. The funding scheme includes 30% of total funding from University of Helsinki and 10% from companies (Affecto, Avaus, Elisa, Fira, Fondia, If, Mehiläinen, Reaktor, Wunder, Wörks).

### Competing interest statement

The authors declare no conflict of interest.

### Additional information

Supplementary content related to this article has been published online at https://doi.org/10.1016/j.heliyon.2020.e04823.

No additional information is available for this paper.

## Supplementary material

The following Supplementary material is associated with this article:

Appendix.pdf Puzzles used in the experiment. Appendix.pdf

## Footnotes

1 https://github.com/coblok/coblok

2 https://github.com/coblok/coblok
