# Supplementary material for: Collaborative block design task for assessing pair performance in virtual reality and reality
Source: Heliyon. 2020 Sep 14;6(9):e04823. doi: 10.1016/j.heliyon.2020.e04823 (PMC7494474; doi:10.1016/j.heliyon.2020.e04823)
Supplement: Appendix.pdf — Puzzles used in the experiment. [file mmc1.pdf]

Appendix A. Puzzles used in the experiment

SET 1

Puzzle	Puzzle cards		Solution	Alternative
1.1	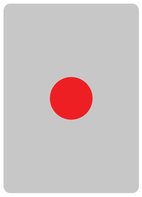	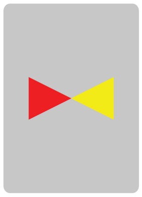	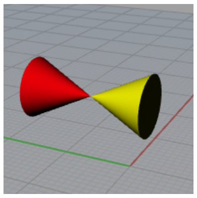	
1.2	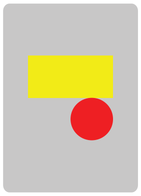	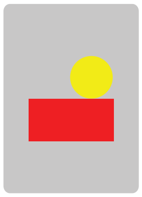	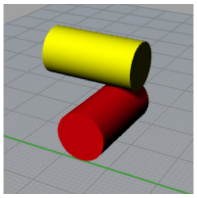	
1.3	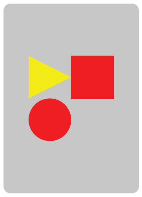	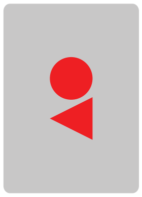	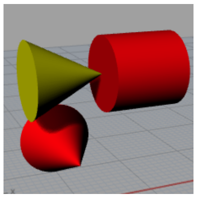	
1.4	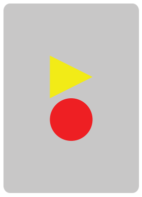	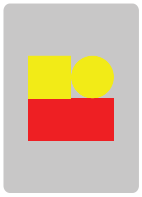	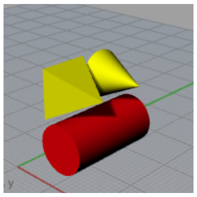	
1.5	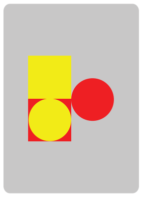	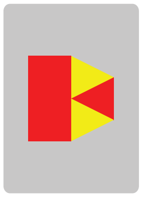	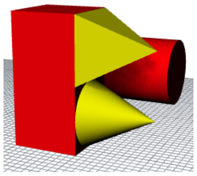	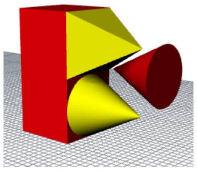

SET 2

Puzzle	Puzzle cards	Solution	Alternative
2.1	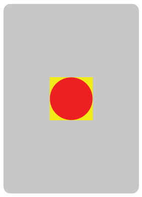 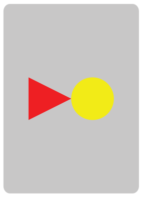	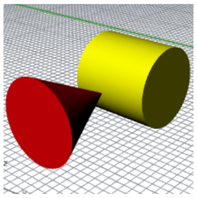	
2.2	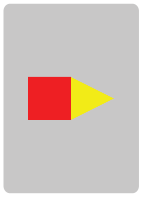 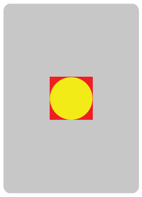	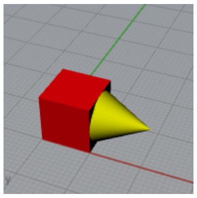	
2.3	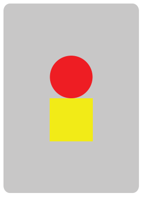 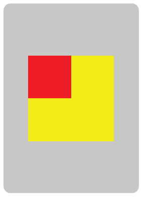	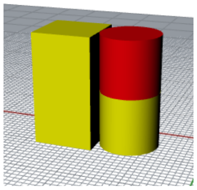	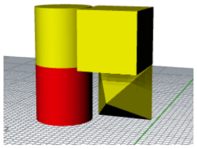
2.4	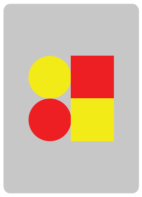 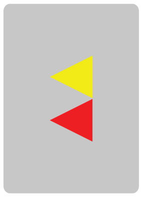	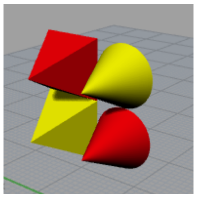	
2.5	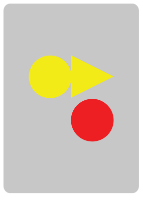 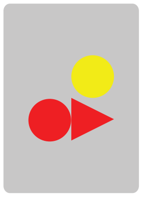	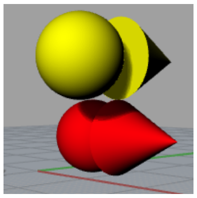	
